# Influence of head models on EEG simulations and inverse source localizations

**DOI:** 10.1186/1475-925X-5-10

**Published:** 2006-02-08

**Authors:** Ceon Ramon, Paul H Schimpf, Jens Haueisen

**Affiliations:** 1 Department of Electrical Engineering, University of Washington, Seattle, WA 98195, USA; 2 School of Electrical Engineering and Computer Science, Washington State University, Spokane, WA 99202, USA; 3 Biomagnetics Center, Department of Neurology, FriedrichSchiller University, Germany and Institute of Biomedical Engineering and Informatics, Technical University Ilmenau, Germany

## Abstract

**Background:**

The structure of the anatomical surfaces, e.g., CSF and gray and white matter, could severely influence the flow of volume currents in a head model. This, in turn, will also influence the scalp potentials and the inverse source localizations. This was examined in detail with four different human head models.

**Methods:**

Four finite element head models constructed from segmented MR images of an adult male subject were used for this study. These models were: (1) Model 1: full model with eleven tissues that included detailed structure of the scalp, hard and soft skull bone, CSF, gray and white matter and other prominent tissues, (2) the Model 2 was derived from the Model 1 in which the conductivity of gray matter was set equal to the white matter, i.e., a ten tissue-type model, (3) the Model 3 was derived from the Model 1 in which the conductivities of gray matter and CSF were set equal to the white matter, i.e., a nine tissue-type model, (4) the Model 4 consisted of scalp, hard skull bone, CSF, gray and white matter, i.e., a five tissue-type model. How model complexity influences the EEG source localizations was also studied with the above four finite element models of the head. The lead fields and scalp potentials due to dipolar sources in the motor cortex were computed for all four models. The inverse source localizations were performed with an exhaustive search pattern in the motor cortex area. The inverse analysis was performed by adding uncorrelated Gaussian noise to the scalp potentials to achieve a signal to noise ratio (SNR) of -10 to 30 dB. The Model 1 was used as a reference model.

**Results:**

The reference model, as expected, performed the best. The Model 3, which did not have the CSF layer, performed the worst. The mean source localization errors (MLEs) of the Model 3 were larger than the Model 1 or 2. The scalp potentials were also most affected by the lack of CSF geometry in the Model 3. The MLEs for the Model 4 were also larger than the Model 1 and 2. The Model 4 and the Model 3 had similar MLEs in the SNR range of -10 dB to 0 dB. However, in the SNR range of 5 dB to 30 dB, the Model 4 has lower MLEs as compared with the Model 3.

**Discussion:**

These results indicate that the complexity of head models strongly influences the scalp potentials and the inverse source localizations. A more complex head model performs better in inverse source localizations as compared to a model with lesser tissue surfaces. The CSF layer plays an important role in modifying the scalp potentials and also influences the inverse source localizations. In summary, for best results one needs to have highly heterogeneous models of the head for accurate simulations of scalp potentials and for inverse source localizations.

## Background

Highly heterogeneous finite element method (FEM) models of the head have recently become increasingly popular for EEG (electroencephalography) simulations and inverse reconstructions of the electrical sources in the cortex. How does the complexity of these models influence the forward and inverse simulations? We have examined this question with four different FEM models of the head varying in complexities from five to eleven tissue-types. In particular, we examined the effects of CSF, gray and white matter on the forward and inverse simulations for the sources located in the motor cortex area. Our results show that both the scalp potentials and the inverse source reconstruction are significantly influenced by the model complexity.

Previous studies with boundary element method (BEM) models of the head have examined how volume currents affect the forward EEG simulations and also their effects on inverse source localizations [1,2]. It was found that a 3-compartment BEM model of the head performed better than a 3-shell spherical model of the head, particularly in basal brain areas, including the temporal lobe [1]. Recently, a five tissue-type FEM model of the head has also been used for MEG (magnetoencephalography) simulations and source reconstructions [3]. That study compared the performance of a five tissue-type FEM model with a spherical head model and found that the five tissue-type FEM model performed better in accounting of the volume currents and in inverse source localization. These previous studies show that more complex head models account for volume currents more precisely as compared to simpler, e.g., spherical, head models. Thus, highly heterogeneous finite element models of the head have a potential to further improve the inverse source localizations. In related studies, a five tissue-type FEM model of the head has also been used for efficient computations of the lead fields [4,5] and also for analyzing the effects of tissue conductivities on MEG forward and inverse simulations [6].

## Methods

**Figure 1:**
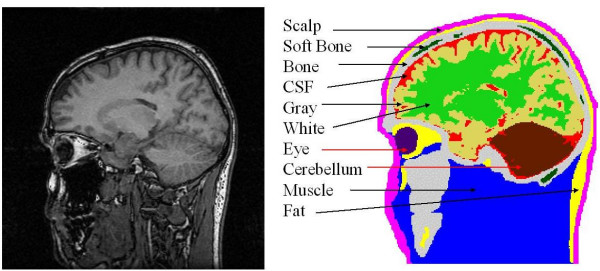
(Left) A raw MRI slice, (right) segmented slice with ten major tissues identified in it. The soft tissue which is present in other slices is not included here.

**Figure 2:**
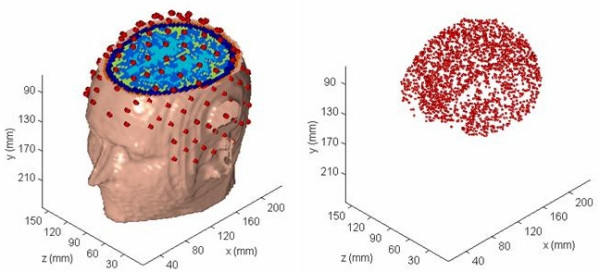
(Left) A-3D view of the head model superimposed with 145 EEG electrode positions, (right) a map of cortical voxel points in the gray matter.

Finite element models of the head were constructed from the segmented MRI (magnetic resonance imaging) slices of an adult male subject. The T1 weighted sagittal MRI slices with 3.2 mm thickness were collected with a 1.5 Tesla GE Signa scanner. The original MR slices were of 256 × 256 resolution with 1.0 mm size pixels [7]. A total of 51 contiguous slices was used. Eleven major tissues were identified in the image slices. A raw MR slice and the segmented slice are shown in Figure 1. The MR images were segmented by use of a semiautomatic tissue classification software developed by us [8]. Our segmentation software has gone through a major revision in 2001 and it is constantly upgraded to include latest technologies for image segmentation. After segmentation, the slices were checked by a radiologist for any errors and the segmentation was corrected as needed. A 3-D view of the head model and the scalp EEG electrode positions are shown in Figure 2. The left figure shows the head model and the scalp electrodes. The right figure is only for the cortical voxel nodes. The EEG electrode positions were generated by starting with the 82 sampling points of an extended EEG 10–20 layout, and visually interpolating an additional 63 points.

For simulation studies, four models were used:

Model 1: Full model with eleven tissue-types,

Model 2: Full model with the conductivity of gray matter equal to white matter, i.e., a ten tissue-type model,

Model 3: Full model with the conductivities of gray matter and CSF equal to the white matter, i.e., a nine tissue-type model,

Model 4: Five tissue model consisting of scalp, hard skull, CSF, gray and white matter.

The eleven tissues used in the Model 1 are: scalp, fat, muscle, hard skull bone, soft skull bone, gray matter, white matter, eyes, cerebellum, cerebrospinal fluid (CSF) and soft tissue.

**Figure 3:**
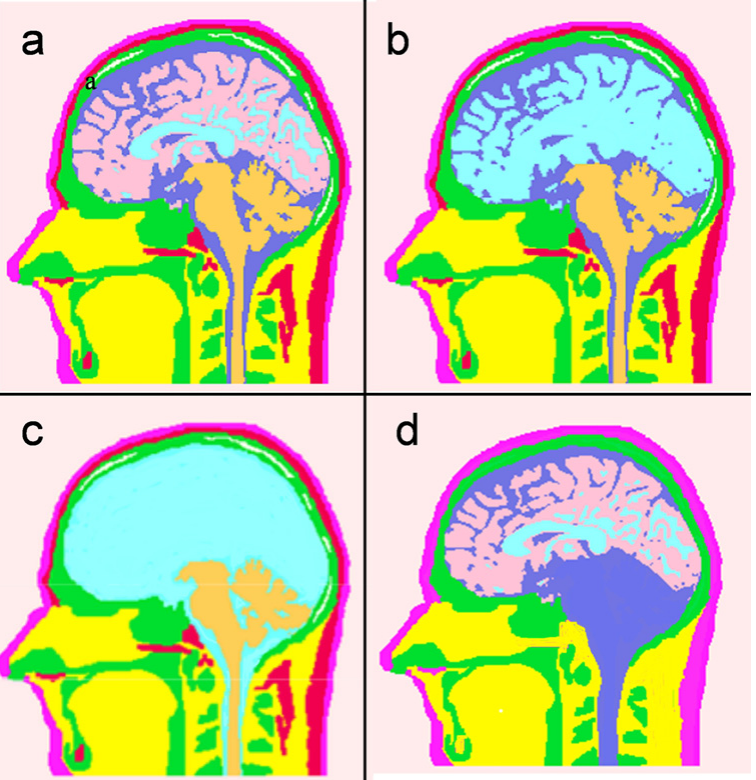
Human head models with varying tissue complexities. (a) Model 1 with eleven tissue-types, (b) Model 2 with distinction between gray and white matter removed, (c) Model 3 with no distinction between CSF regions, white matter and gray matter, (d) Model 4 with five major tissue-types.

The Model 4 is composed of fewer tissue-types as compared with other models. The major tissues in this model are: scalp, hard skull, CSF, gray and white matter. This model was developed by replacing the other tissues in each slice with the nearby tissues. As an example, soft bone was replaced with the hard bone in the skull; cerebellum was replaced with CSF; fat layer near to the scalp was replaced with the scalp and eye sockets were replaced with soft tissue. Similarly, all tissues below the jaw in the Model 4 were treated as soft tissue while building the FEM model of the head. The Model 4 is similar to van Uitert's model [3]. They call it a six tissue model by including external air as one of the tissues. Refer to Figure 3 for details of these four models. Figure 3a is for the Model 1. It has all the tissue surfaces intact. In Figure 3b for the Model 2, distinction between the gray and white matter boundaries has been eliminated. Similarly, in Figure 3c for the Model 3, there is no distinction between the CSF, gray and white matter tissue boundaries. The Model 4 is shown in Figure 3d. It has no soft skull bone, cerebellum and the fat layer.

The segmented images were subsampled to a 2 × 2 mm resolution for building finite element models of the head. All four models had a voxel resolution of 2 × 2 × 3.2 mm. The voxels were hexahedral, i.e., brick-shaped elements with linear basis functions. There were 835,584 hexahedral voxels and 865,332 nodes for the Model 1. The tissue resistivity values used in the models are given in Table 1. These values have been used by us before in our head modeling studies [7] and are compiled from published values [9-11]. Using an uniform finite element solver [12,13], the lead fields at 145 EEG electrode positions were computed for all four models due to dipolar sources in the motor cortex area. The motor cortex within a volume of 3 cm cube was represented by 716 hexahedral voxels. The lead fields were computed for *x*, *y *and *z *orientations of the dipoles. This was done by placing one dipole at a time at a node of the voxel and the scalp potentials were computed. The dipole magnitude was 100 *μA-meter*. These dipoles were represented with an approximate Laplace formulation described elsewhere [14,15]. The lead field for a combined dipole in a voxel was obtained by summing up the lead fields of the individual *x*, *y *and *z *oriented dipoles in that particular voxel. Since the dipole magnitudes are same in all three directions, the orientation of the combined dipole is at 54.7° from any of the *x*, *y *and *z *axis. This angle was computed by use of direction cosine law.

**Table 1:** Head tissue resistivity and conductivity values.

**Tissue**	**Resistivity (Ohm cm)**	**Conductivity (Siemens/cm)**
Brain White Matter	700	1.428E-3
Brain Gray Matter	300	3.334E-3
Spinal Cord and Cerebellum	624	1.6026E-3
Cerebrospinal Fluid (CSF)	65	15.38E-3
Hard Bone	16000	6.25E-5
Soft Bone	2180	4.587E-4
Muscle	900	1.1112E-3
Fat	2500	4.0E-4
Eye	198	5.0505E-4
Scalp	230	4.3478E-3
Soft Tissue	576	1.7361E-3

For the inverse source localizations, first the forward problem was solved. The EEGs were simulated at 145 scalp electrodes using the Model 1. A set of 716 trial inverse runs was made covering the 3 cm cube motor cortex area. For each trial, a dipolar source with random magnitude was placed at a given position in the motor cortex and the scalp potentials were computed. Uncorrelated Gaussian noise was added to achieve the desired signal to noise (SNR) ratio. The SNR was defined as [16]:



where *var*(*V*_*exact*_) is the variance of the simulated noisefree observations, and σ^2 ^is the variance of the added noise.

These forward simulated EEGs were then used for inverse source localizations using the lead fields of four different models. The Model 1 was used as a reference model. Inversions were performed with the least-squares technique. An exhaustive search pattern was used, i.e., inversion was performed for each possible source location in the motor cortex and the site producing the smallest residual norm was selected as the best possible source location.

All computations were performed on an Intel 3.2 GHz workstation with 1.2 gigabytes memory. Each run for the lead field computation took between 2–3 seconds. Post-processing and visualizations were done using the Matlab software, version 7.1 (Mathworks, Inc., Natick, MA).

## Results

### Forward simulation

**Figure 4:**
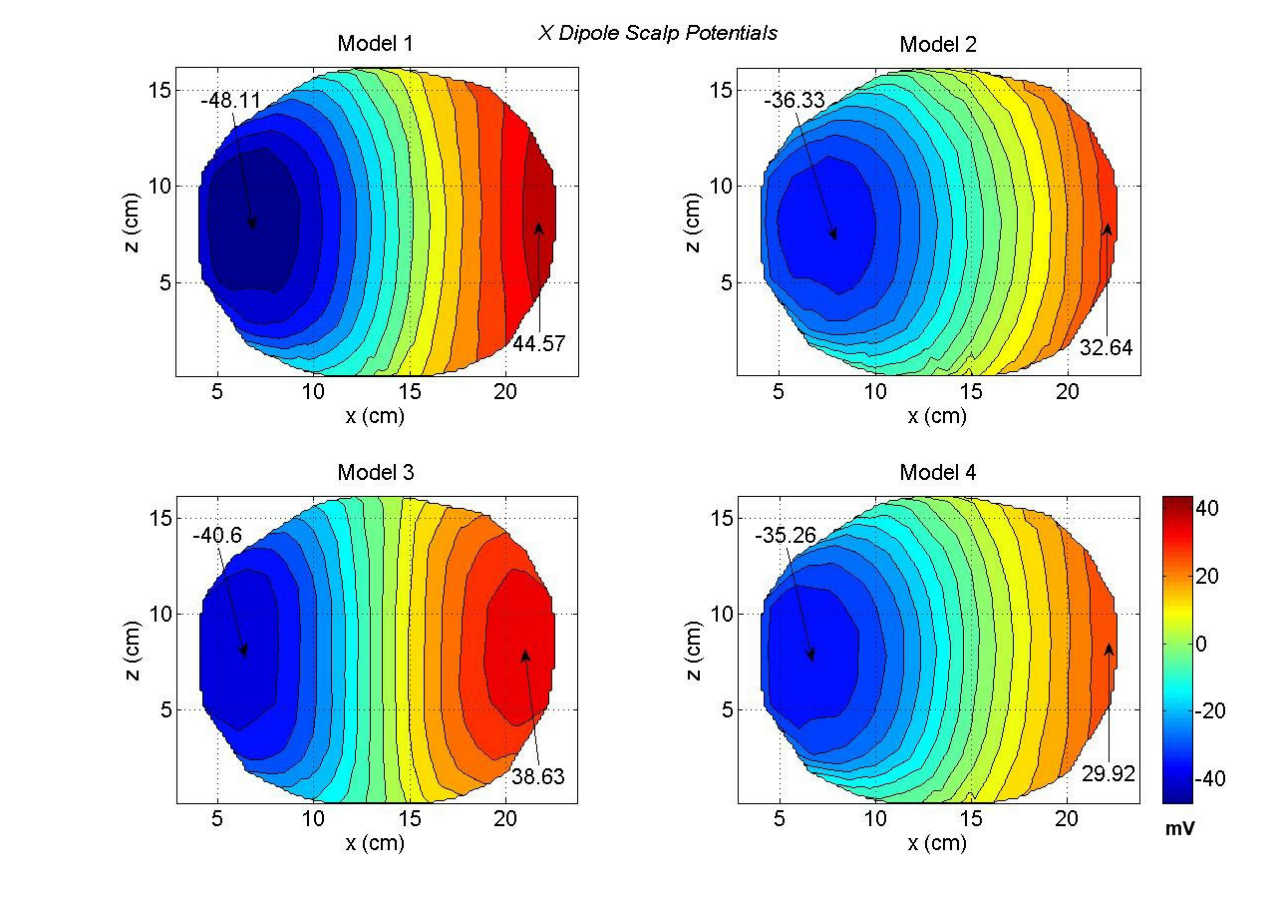
Scalp potentials of four head models for an *x *oriented dipole in the motor cortex area. All values are in *mV*. The Model 1 has the largest negative and positive peaks. The positive and negative contours for the Model 3 are very symmetrical and the zero crossing line is almost vertical.

The contour plots of scalp potentials for an *x *oriented dipoles of all four models are shown in Figures 4. These contour plots are for a typical *x *oriented dipole in the motor cortex. This particular dipole was located at a depth of 5 cm from the scalp surface in the motor cortex. The *x *coordinate increases from anterior (front) to the posterior (back) of the subject and the *z *coordinate increases from left to the right side of the subject. Refer to Figure 2 for the coordinate orientation. The magnitude scale is same for all four plots and it is included in the bottom right contour plot. The magnitude values are in milli-volts (*mV*) in all of the plots. We have followed the same procedure for the magnitude scale in other figures for the contour plots. The zero-crossing line gradually becomes vertical as one progressively moves from the full model (Model 1) to the less tissue-type models, e.g., Model 2 to Model 3. The difference between the two models is that Model 3 eliminates the distinction between the CSF and brain tissue contained in Model 1. The relative locations of the maximum and minimum contour peaks are very symmetrical for the Model 3 as compared to the other models. The Model 4 lacks the details of scalp fat, muscle, and skull anisotropy due to the soft skull bone. Due to these tissue related differences, there are noticeable changes in the scalp potentials of the Model 4 as compared with the full model, i.e., Model 1.

**Figure 5:**
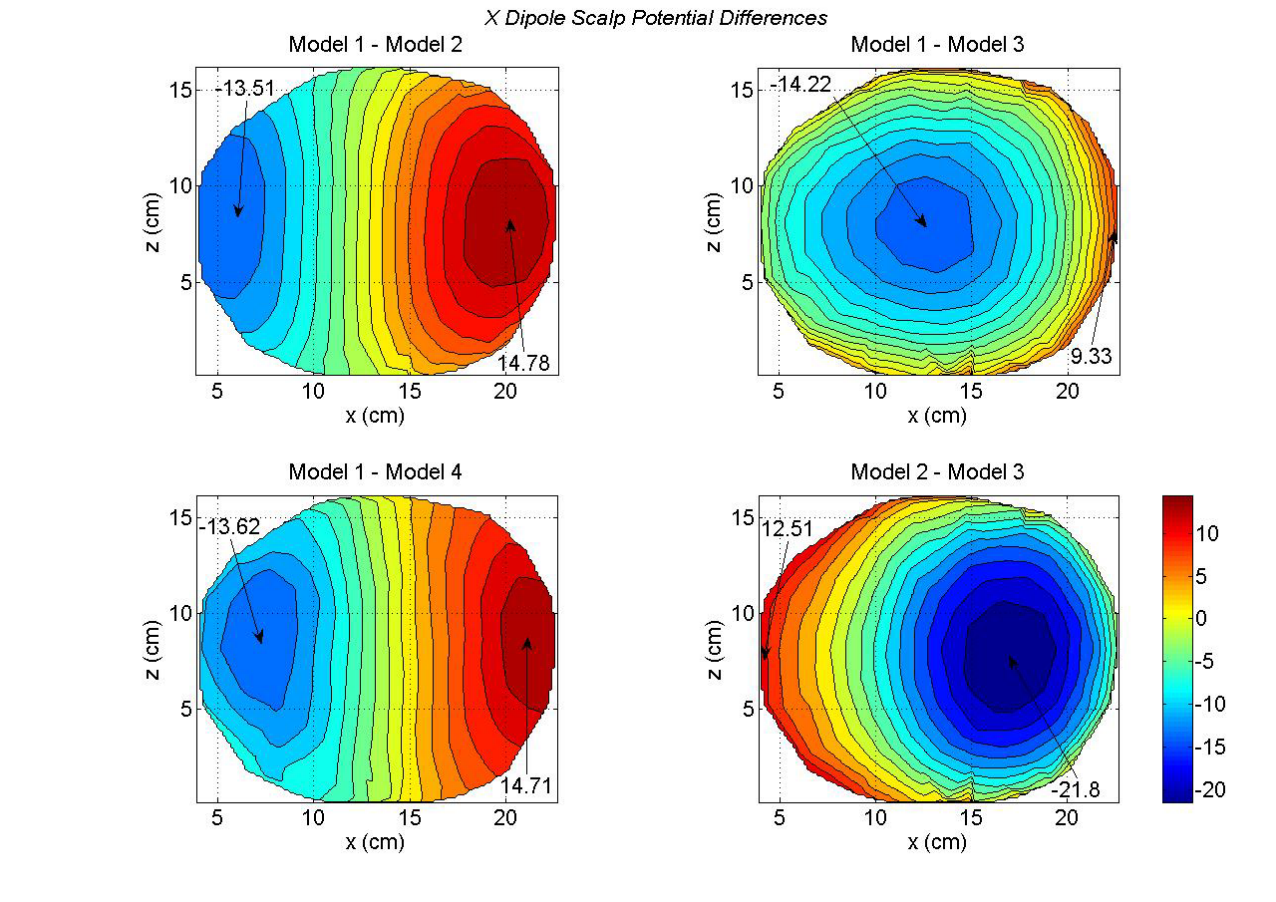
Differences in scalp potentials of head models for an *x *oriented dipole in the motor cortex area. The contours for the Model 1 – Model 2 (top left plot) are very symmetrical and exhibit a dipolar pattern. The negative contours for the Model 1 – Model 3 (top right plot) dominate the plot. The Contour plots for the Model 1 – Model 4 (bottom left plot) are also symmetrical and exhibit a dipolar pattern. The contours of the Model 2 – Model 3 are also dominated by negative contours.

These differences are more visible in the scalp potential difference plots given in Figure 5. The Model 1 is used as a reference model. There are some noticeable differences between the Model 1 and Model 2 (top left plot). These differences are very significant for the Model 1 and Model 3 (top right plot). This is expected because the difference between the brain matter and the CSF has been eliminated in the Model 3. This implies that the CSF plays an important role in redistributing the volume currents and thereby modifying the scalp potentials [17]. The differences between the Model 1 and Model 4 are shown in the bottom left plot. The difference in negative and positive peak values are -13.62 and 14.71 *mV*. The differences between the Model 2 and Model 3 are shown in the bottom right plot. Once again it highlights the effect of CSF on scalp potentials. The Model 3 lacks the details of CSF boundaries which are included in the Model 1.

**Figure 6:**
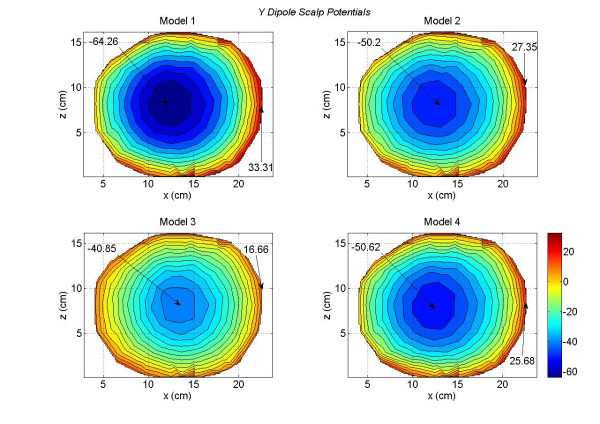
Scalp potentials of four head models for a *y *oriented dipole in the motor cortex area. The *y *axis points downward from top to the bottom of the head. The Model 1 has the largest negative peak value.

**Figure 7:**
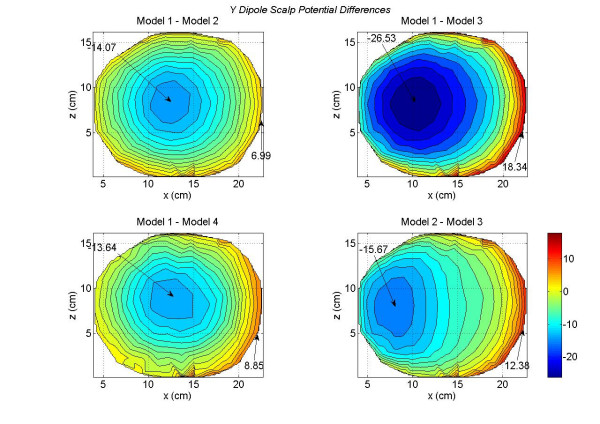
Differences in scalp potentials of head models for a *y *oriented dipole in the motor cortex area. The Model 1 – Model 3 (top right plot) has the largest negative peak value.

The contour plots of scalp potentials for a *y *oriented dipole are shown in Figure 6. This dipole was located at the same voxel node where the *x *oriented dipole was located. The *y *axis is pointing downward from the top of the head as shown in Figure 2. The primary dipolar current exists from the voxel in the motor cortex and travels toward the middle and lower part of the brain. The returning volume currents enter in the voxel from the top. This is the reason that negative peak is most prominent in Figure 6. The magnitude of the negative peak is largest (-64.26 *mV*) for the Model 1 and lowest (-40.85 *mV*) for the Model 3. There is more uniformity in the distribution of positive scalp potentials (reddish color) for the Model 3 (bottom left plot) as compared to the other plots. For Models 1, 2 and 4, the positive scalp potentials are more tilted toward the right side of the plots. The difference plots are given in Figure 7. These differences are very significant between the Model 2 and 3 as shown in the bottom right plot of Figure 7. Once again, these are due to the lack of the CSF layer in the Model 3.

**Figure 8:**
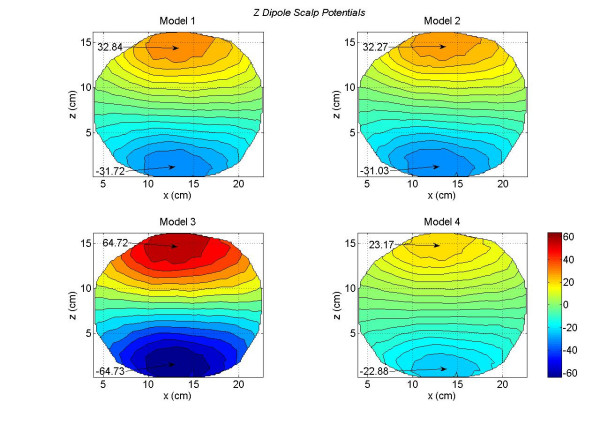
Scalp potentials of four head models for a *z *oriented dipole in the motor cortex area. The contour plots exhibit a dipolar pattern. The Model 3 has the largest positive and negative peaks which could be related to the lack of CSF in the Model 3.

**Figure 9:**
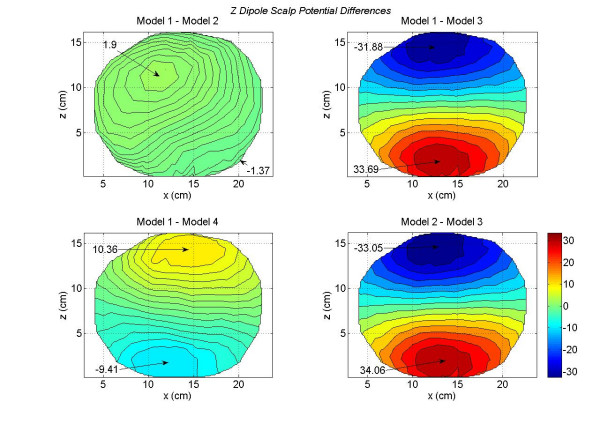
Differences in scalp potentials of head models for a *z *oriented dipole in the motor cortex area. The top right plot and the bottom right plot show large peak values.

For a *z *oriented dipole, the contour plots of all four models are given in Figure 8. This *z *oriented dipole was located at the same voxel node where the *x *and *y *oriented dipoles were located. The location of the maximum and minimum peaks are well defined for all four models. One interesting feature to note is that the peak values for the Model 3 are twice in magnitude as compared to the Model 1. This also could be attributed to the lack of CSF in Model 3. Also the zero-crossing line gradually becomes horizontal as one progresses from Model 1 to Model 2 and then to Model 4. There are noticeable differences between the contour plot of the Model 1 (top left) and the contour plot of the Model 4 (bottom right). In particular, note the differences between the peak values. These model dependent differences in the scalp potentials are plotted in Figure 9. The significant differences are between the Model 1 and Model 3 (top right) and the Model 2 and Model 3 (bottom right). These differences, most likely, are due to the lack of CSF in Model 3. Also, note the 180 degree shift in the location of the contour peaks. In the Figure 8, the positive contour peaks are on the top for all four models. In difference plots, the Model 1 – Model 3 peaks (top right) and the Model 2 – Model 3 peaks (bottom right) are reversed as compared with Figure 7. This is due to the large peak values for the Model 3 in Figure 8. The scalp potential differences are also noticeable for the Model 1 – Model 4 (bottom left plot in Figure 9). The differences in peak values are 10.36 *mV *and -9.41 *mV*; which are significant.

**Figure 10:**
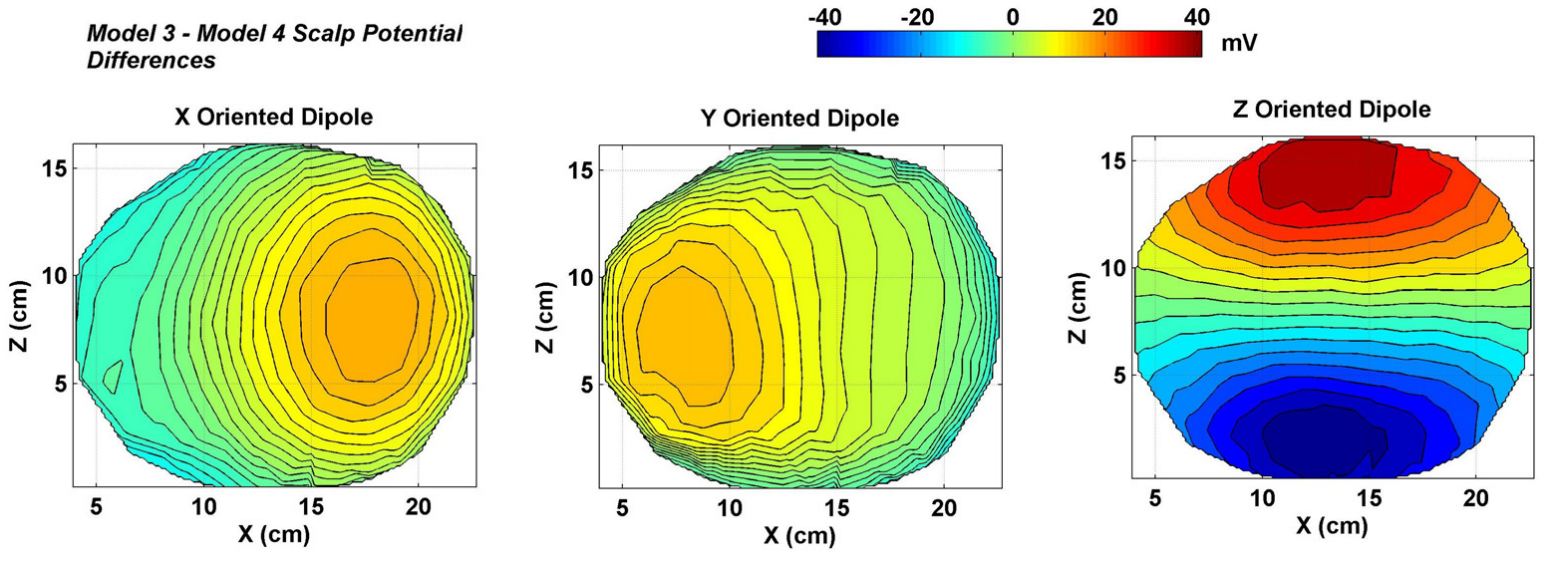
Differences in scalp potentials for the Model 3 and 4. The scalp potential differences for the *z *oriented dipole (right plot) are very large as compared to the *x *or *y *oriented dipole.

The scalp potential differences between the Model 3 and the Model 4 are of special significance and are displayed in Figure 10. These plots are for the *x*, *y *and *z *oriented dipoles in the motor cortex area. The Model 3 does not have CSF and the Model 4 includes the CSF layer. These differences will help in quantifying the effects of CSF on scalp potentials. For an *x *oriented dipole the contour plot (left plot in Figure 10) shows that the differences are higher on the right side of the figure with a peak value of 8.71 *mV*. This difference is also visible in the contour plots of scalp potentials (Figure 4) of the Model 3 and 4 for an *x *oriented dipole. This is for the positive valued contours that are shown in reddish color in Figure 4. The negative contours are shown in bluish color in Figure 4. The difference in negative contour values between the Model 3 and 4 from the Figure 4 is -5.34 *mV*. This is also visible in the left plot of Figure 10 for an *x *oriented dipole. Similarly for a *y *oriented dipole, the scalp potentials are given in Figure 6 and the difference of scalp potentials between Model 3 and 4 is given in the middle plot of Figure 10. The Model 3 has a larger negative peak as compared to Model 4. Due to this in the difference plot (Figure 10) it comes out as a positive peak of 9.77 *mV*. For the *x *and *y *oriented dipoles, these differences are less as compared to the *z *oriented dipole in Figure 10. These difference plots of Figure 10 suggest that the CSF layer influences scalp potentials very significantly for a *z *oriented dipoles as compared with an *x *or a *y *oriented dipole.

### Inverse results

**Figure 11:**
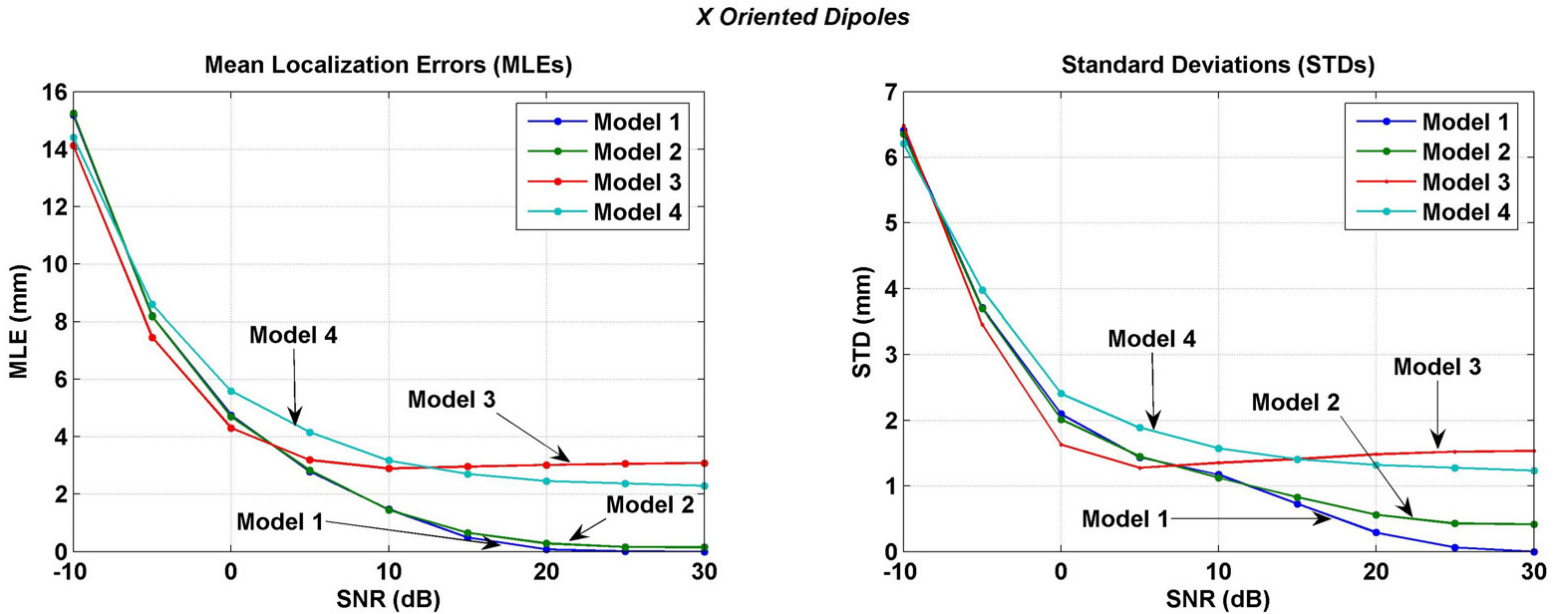
Mean localization errors (MLEs) and standard deviations (STDs) of four models for an *x *oriented dipole in the motor cortex area. (Left) mean localization errors and (right) standard deviations. To read the values, use both plots simultaneously. As an example, at 0 dB of SNR, the MLE ± STD is 4.30 ± 1.63 for the Model 3. Similarly, the MLE ± STD for other models and at different values of SNR can be extracted.

**Figure 12:**
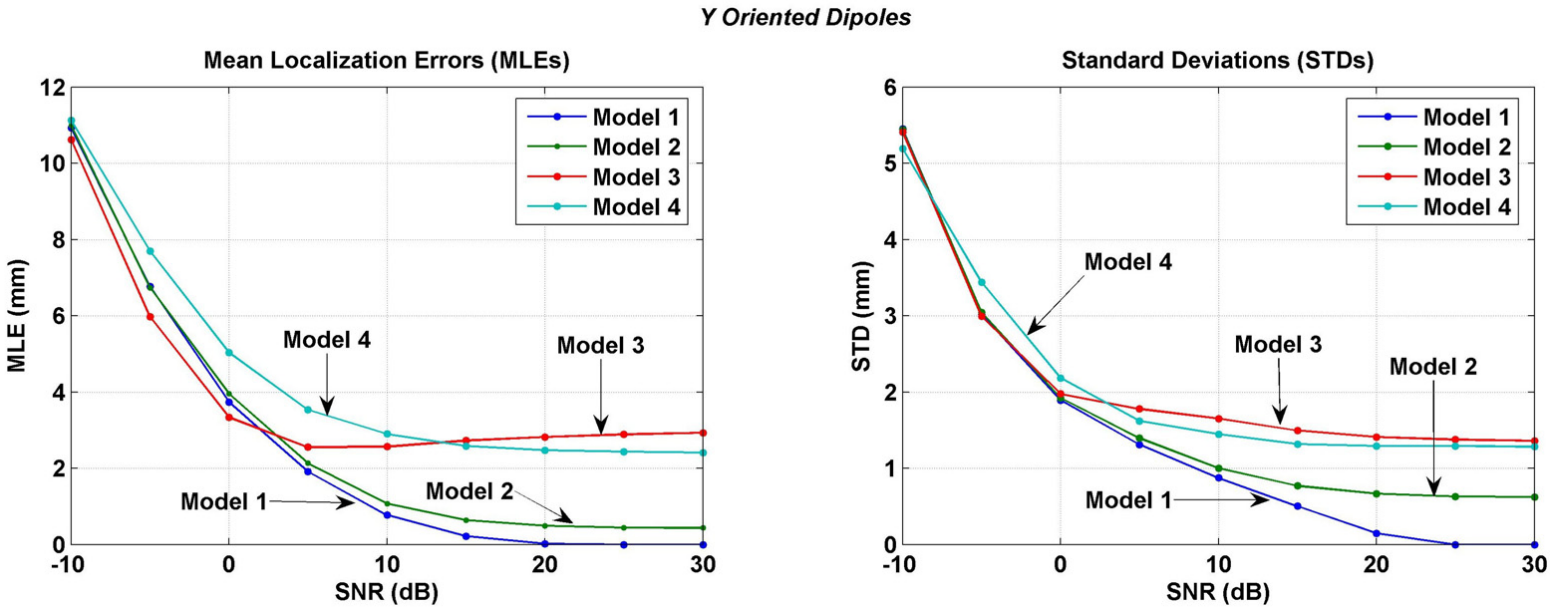
Mean localization errors (MLEs) and standard deviations (STDs) of four models for a *y *oriented dipole in the motor cortex area. (Left) mean localization errors and (right) standard deviations.

**Figure 13:**
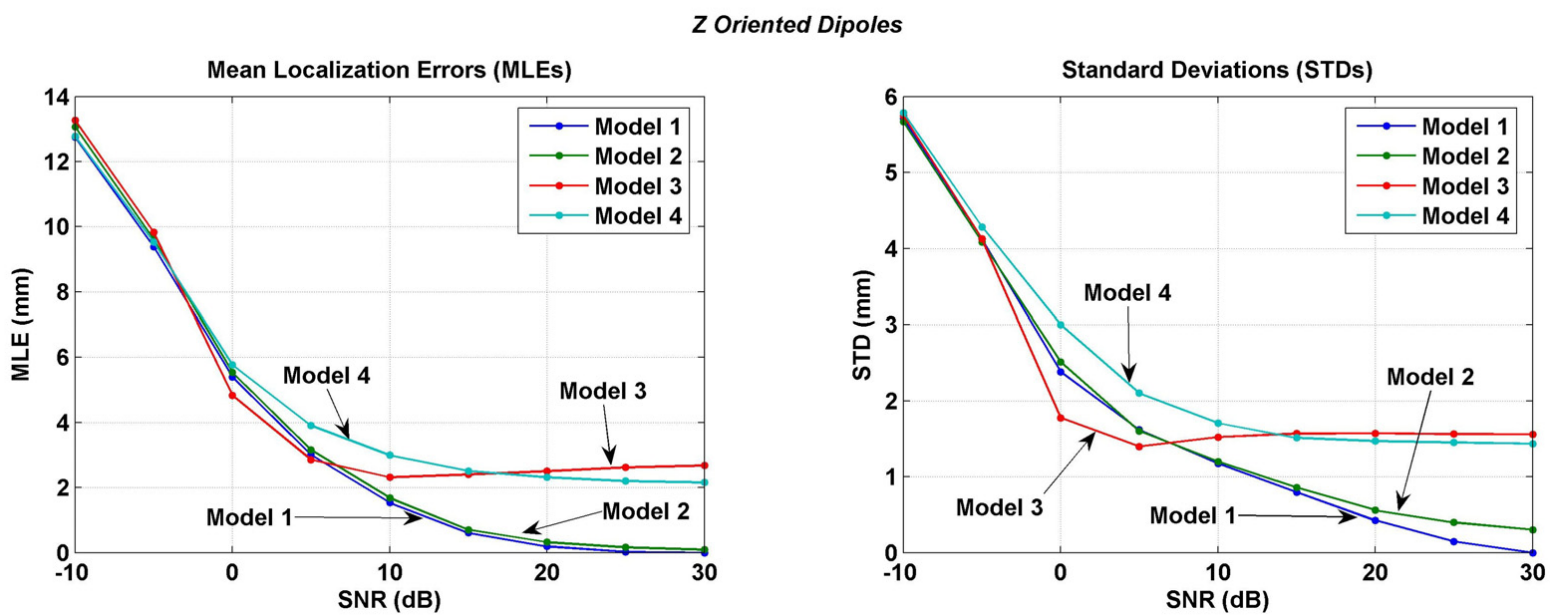
Mean localization errors (MLEs) and standard deviations (STDs) of four models for a *z *oriented dipole in the motor cortex area. (Left) mean localization errors and (right) standard deviations.

**Figure 14:**
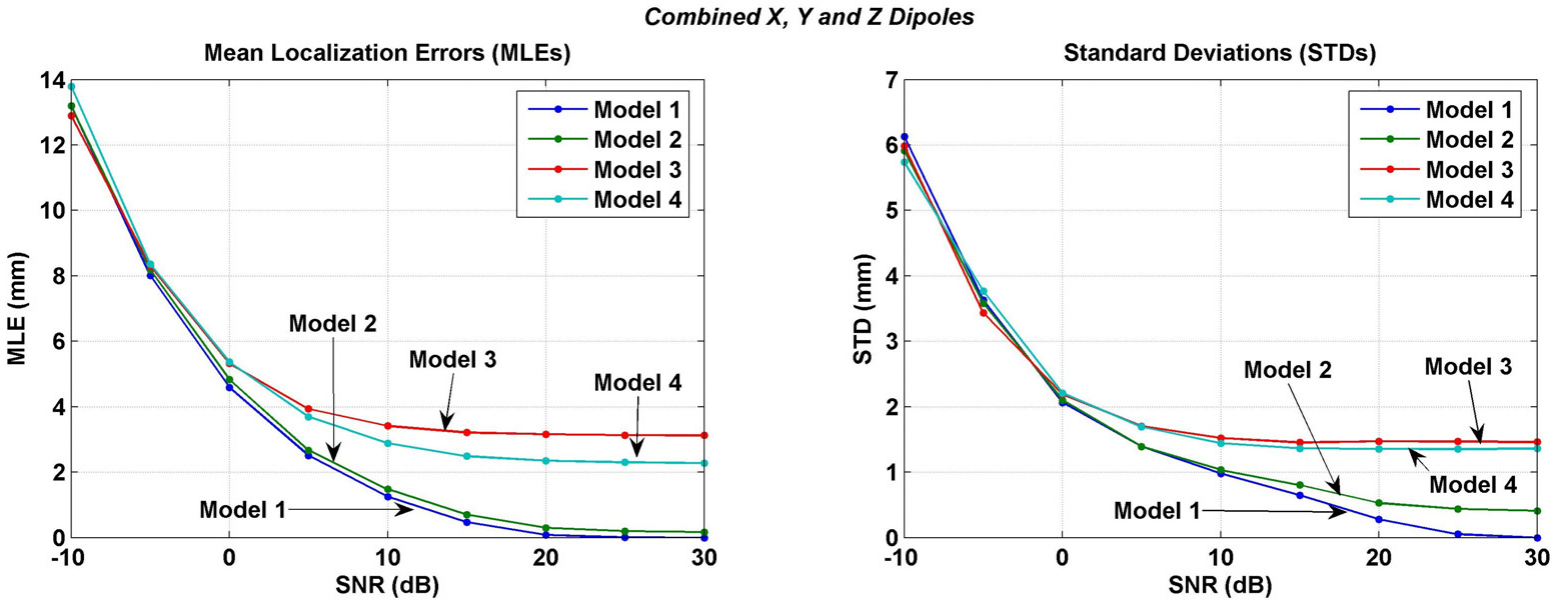
Mean localization errors (MLEs) and standard deviations (STDs) when inverse analysis was performed with combined lead fields of *x*, *y *and *z *oriented dipoles in the motor cortex area. The Model 3 which does not have CSF layer consistently performs worse than the other models for SNR values in the range of 0 to 30 dB.

Mean localization errors (MLEs) and standard deviations (STDs) averaged over 716 trials covering all the possible dipole locations in a 3 cm cube motor cortex are plotted in Figures 11 to 14. MLEs and STDs for the *x *oriented dipoles are given in Figure 11, for the *y *oriented dipoles are given in Figure 12 and for the *z *oriented dipoles are given in Figure 13. The STD values are large. It was difficult to plot them as vertical error bars in the MLE plots because one error bar was overshadowing the other and thereby making it difficult to interpret the results. Because of this, the MLEs and STDs are plotted separately. In these figures, left plot is for MLEs and the right plot is for STDs. To read the values, one has to use both plots simultaneously. As an example in Figure 11 at 0 dB of SNR, the MLE ± STD is 4.30 ± 1.63 for the Model 3. Similarly, the MLE ± STD for other models and at different values of SNR can be extracted.

All models have similar MLEs for SNR of -10 to 0 dB. After that differences in MLEs for different models begin to emerge. For all three dipole orientations, Model 1 and Model 2 have the lowest errors in the SNR range of 0 to 30 dB. The Model 3 behaves in a peculiar fashion. MLEs decrease with increasing SNR from -10 to 5 dB. After that for all three dipole orientations the MLEs slightly increase with increasing SNR in the range of 3 to 30 dB. This could be because the Model 3 does not have any morphological distinction between the CSF, gray and white matter and CSF plays an important role in redistributing the volume currents [17]. Errors, i.e., MLEs for the Model 4 are higher as compared to the Model 1 or Model 2. This could be due to the lack of muscle, fat and the skull bone anisotropy in the Model 4 which are included in the Model 1 or Model 2.

Results of the inversions performed with the combined lead fields of *x*, *y *and *z *oriented dipoles are given in Figure 14. This is not an average of the results shown in Figures 11, 12, and 13. These results were obtained by using the combined lead fields of all three, *x*, *y *and *z *orientations of the dipoles. Here the Model 3 has consistently largest error as compared to other models for SNR values in the range of 0 to 30 dB. This is different as compared to the behavior of the Model 3 in Figures 11, 12, 13. The MLEs for the Model 4 are still larger than the Model 1 or the Model 2.

In all of the inversion results given in Figures 11, 12, 13, 14, the STD values are in the range of 6.5 to 5.4 mm at the SNR of -30 dB. The STD values decrease for SNR values from -30 to 0 dB. The behavior of all four models is very different for the SNR values in the range of 0 to 30 dB. For the *x *oriented dipoles (Figure 11), the STD values for the Model 3 slightly increase after the SNR of 5 dB. The same pattern is also present for the *z *oriented dipoles (Figure 12). The Model 4 has higher STD values as compared with the Model 1 or Model 2.

## Discussion

These results suggest that head model complexities influence both the forward EEG simulations and the inverse source localizations. The above results also suggest that the Model 3 has larger source localization errors as compared to the full model, i.e., Model 1. In Model 3, the difference between the CSF and brain matter was eliminated. The resistivity of the CSF is less than the gray and white matter. So in the full model, i.e., Model 1, the currents will follow the structural paths of CSF channels in the brain. In Model 3 this distinction does not exist and the spread of the currents will be more uniform as compared to the Model 1 and 2. This will change the scalp potentials over a large portion of the scalp surface. It has been shown earlier that CSF plays an important role in distributing the currents in a head model [17]. This could also be the reason that Model 3 performs much worse in inverse source localizations as compared to Model 1 or Model 2. The electrical conductivity of the human CSF is well documented in the literature [18] and can be incorporated in head models.

These model dependant results should also be compared with the tissue conductivity related results where one changes the tissue conductivity in steps and examines the changes in the scalp potentials [19-22]. Previous studies have not eliminated tissue boundaries, but they have used incremental changes in the tissue conductivities or have used upper and lower bounds of the tissue conductivity values [6,21]. Also, detailed scalp potential maps are not available in previous studies to perform a comparative analysis. In general, previous studies have found that both the forward and inverse results are severely influenced by changes in the conductivity of skull bone, CSF, gray and white matter. In particular, conductivity of skull bone [6,21-23] and the skull anisotropy [20] severely influences the EEG and MEG simulations and inverse source reconstructions. Conductivity related inverse localization errors could be of the order of 2.35 mm to 9.59 mm [6]. Our results also show that localization errors increase as the complexity of the model decreases. The fat, muscle and soft bone structures are not included in the Model 4 and this model has larger source localization errors as compared to the Model 1 or Model 2. This suggests that highly heterogeneous finite element models of the head are needed to reduce the source localization errors. Our work here was limited to dipoles in the motor cortex area. However, one could expect similar results for dipoles located in other parts of the cortex.

As our results show that CSF layer plays an important role. It influences simulations of scalp potentials and also influences the inverse source localizations. The CSF layer is difficult to segment in T1 or T2 weighted MR images. One needs to be aware that miss-segmentation of the CSF layer could become a source of error in EEG computations. There is also a related issue regarding the position of the brain and the CSF layer. The MRI data is collected while the subject is in a supine position and the EEG data is collected while the subject is, generally, in a sitting position. There could be a slight difference in the location of the brain within the skull when a subject is in supine position as compared to when the subject is sitting. The CSF layer could also be slightly shifted due to a shift in the brain position. This shift in the brain position between the supine and sitting position is an additional source of error which is difficult to account for while building the computer models of the head.

Here we have used an exhaustive search pattern to localize the sources. This means that all the possible nodes were searched in the 3 cm cubic volume of the motor cortex. The node producing the least error was selected as the possible source location. This provides the best behavior of a given model in inverse source localizations. The reported mean localization errors are the best results one could expect from a given model.
